# MicroRNA-155 regulates casein kinase 1 gamma 2: a potential pathogenetic role in chronic lymphocytic leukemia

**DOI:** 10.1038/bcj.2017.80

**Published:** 2017-09-08

**Authors:** T Zhang, J K Davidson-Moncada, P Mukherjee, R R Furman, E Bhavsar, Z Chen, P Hakimpour, N Papavasiliou, W Tam

**Affiliations:** 1 Division of Hematopathology, Department of Pathology and Laboratory Medicine, Weill Cornell Medicine, New York, NY, USA; 2 Laboratory of Lymphocyte Biology, The Rockefeller University, New York, NY, USA; 3 Epigenomics Core Facility, Weill Cornell Medicine, New York, NY, USA; 4 Division of Hematology and Oncology, Department of Medicine, Weill Cornell Medicine, New York, NY, USA; 5 Department of Healthcare Policy & Research, Weill Cornell Medicine, New York, NY, USA

Chronic lymphocytic leukemia (CLL) is the most common leukemia of adults in Western countries. There are approximately 19 000 new cases diagnosed in the United States in 2016 (https://seer.cancer.gov/statfacts/html/clyl.html). MicroRNAs (miRs) have been implicated as one of the key contributors in the pathogenesis of CLL. One of these miRNAs is miR-155, which is probably the best-characterized miRNA involved in B-cell maturation and function. MiR-155 has a critical role in normal immune function,^1^ including innate response, regulation of the germinal center response and formation of class-switched plasma cells and negative regulation of activation-induced cytidine deaminase. However, miR-155 is also an oncogenic miRNA overexpression of miR-155 in mice or its precursor *BIC* in chickens led to the development of lymphomas. Furthermore, abnormal expression of miR-155 has been observed in a number of lymphoid malignancies, including diffuse large B-cell lymphoma, classical Hodgkin lymphoma, primary mediastinal B-cell lymphoma and CLL.^2^ In various studies, miR-155 has been found consistently overexpressed in CLL cells compared with normal B cells. Although there does not appear to be definitive correlations between miR-155 expression levels and individual CLL prognostic factors, high pretreatment levels of miR-155 in CLL cells or in plasma have been demonstrated to be associated with shorter need-for-treatment interval and failure to achieve complete response, respectively.^3, 4^ The association of adverse clinical outcome in CLL with high miR-155 levels appears to be linked to the capacity of miR-155 to enhance the sensitivity of CLL cells to B-cell receptor ligation.^5^ Thus it is likely that miR-155 has important roles in the pathobiology of CLL.

Identification of targets for miR-155 will facilitate the understanding of how its deregulation contributes to the pathogenesis of CLL. To identify physiologically relevant targets of miR-155 in CLL, we measured miR-155 levels of purified CLL B cells from the peripheral blood of 38 patients using quantitative reverse transcriptase-PCR, as well as 5 sets of naive B cells and 6 sets of memory B cells isolated from hyperplastic tonsils. The results were ranked according to miR-155 levels and *IGVH* status (Supplementary Figure S1). MiR-155 levels in CLL with high miR-155 expression (11 in total) are about fivefold that of CLL with low miR-155 expression (11 in total). The six top-ranked and five bottom-ranked CLLs in each of the *IGVH*-unmutated and -mutated subgroups were selected based on miR-155 expression for global cDNA expression microarray analysis (supplementary Table S1) using Affymetrix HG-U133 Plus 2.0 microarrays (Cat no. 900466, Affymetrix Inc., Santa Clara, CA, USA) containing >54 000 probe sets covering >20 000 characterized human genes. A comparison between the expression profiles of CLL with high miR-155 vs low miR-155 using significance analysis of microarray analysis (5% false discovery rate) revealed 10 probe sets derived from 8 genes that are downregulated (Supplementary Table S2). Three of these genes: *CSNK1G2*, *ZNF652*, and *KLF3*, harbor evolutionarily conserved miR-155 binding sites according to prediction by TargetScan (v 7.1, June 2016). To further validate *CSNK1G2* as a miR-155 target, we generated luciferase reporter plasmids with human *CSNK1G2* 3′-untranslated region containing either wild-type or mutated miR-155-binding site to perform luciferase reporter assays. The results confirmed that miR-155 could repress the reporter gene expression in 293 T cells and that this repressive effect could be relieved by mutation in the seed region of the miR-155-binding site (Supplementary Figure S2).

Within the original cohort, CLLs with low miR-155 had significantly higher levels (~1.6-fold) of *CSNK1G2* mRNA compared with CLLs with high miR-155 (Figure 1a), which validates the results of microarray analysis and is consistent with a negative regulatory effect of miR-155 on *CSNK1G2* mRNA levels. For additional validation, a cohort of 43 additional CLL samples were tested Supplementary Table S3. We did not detect any significant correlation between the miR-155 levels and *CSNK1G2* mRNA expression levels for the new cohort when the entire cohort was analyzed (Figure 1b). When *CSNK1G2* mRNA expression levels were compared between the subgroup of six patients with the highest miR-155 levels and the subgroup of six patients with the lowest miR-155 levels, the high miR-155 subgroup showed a trend of lower *CSNK1G2* levels (Figure 1c). The difference was not statistically significant (*P*=0.17). However, when the cohort was subdivided into *IGVH*-unmutated and -mutated groups, we found a moderate correlation (*R*^2^=0.42) between miR-155 and *CSNK1G2* mRNA levels for the *IGVH*-mutated group but not for the *IGVH*-unmutated group, and the *CSNK1G2* mRNA expression was significantly higher for the low miR-155 expression in the *IGVH*-mutated subgroup (*P*=0.04, Figures 1d–g). We were unable to determine whether similar negative correlation can be seen between miR-155 and *CSNK1G2* protein, as western blotting of protein extracts isolated form primary CLL cells using commercially available antibodies has been unsuccessful in detecting specific bands despite repeated attempts.

We also enforced miR-155 expression in CLL cells to determine its effect on *CSNK1G2* expression. MiR-155 mimic (Cat. no. 472490-001, Exiqon, Vedbæk, Denmark) was introduced into fresh purified CLL B cells from the peripheral blood of patients with *IGVH* mutation and relatively low miR-155 levels. A 1.6-fold Increase in miR-155 resulted in reduction of *CSNK1G2* and *SOCS1* mRNA (as positive control) levels by 17% and 31%, respectively, compared with CLL cells transfected with negative control microRNA (miRCURY LNA microRNA mimic Negative Control 4, Exiqon, Cat. no. 479903-001) (Figures 2a and b). These findings provided direct experimental evidence of a role of miR-155 in inhibiting *CSNK1G2* expression, particularly in *IGVH*-mutated CLL cells.

Our findings suggest that *CSNK1G2* is a physiologically relevant miR-155 target in CLL cells. Previously, *CSNK1G2* has been predicted to be a miR-155 target in diffuse large B-cell lymphoma based on transcriptome profiling^6^ and also by Argonaute-2 RNA immunoprecipitation followed by next-generation sequencing in 293 T cells with enforced miR-155 expression.^7^ CSNK1G2 is an isoform of casein kinase I, an evolutionarily conserved family of serine/threonine kinases implicated in multiple cellular functions, such as differentiation, proliferation and chromosomal segregation. One of the targets of CSNK1G2 is the collagen type IV alpha 3 binding protein (COL4A3BP/CERT), phosphorylation of which can lead to its inactivation and consequent downregulation of endoplasmic reticulum-to-Golgi transport of ceramide and sphingomyelin synthesis.^8^ CSNK1G2 may regulate apoptosis in CLL cells through phosphorylation of CERT and regulation of sphingolipid metabolism.^9^

Recently, Teng *et al.*^10^ reported that miR-155 mimic enhanced both mRNA and protein levels of CD49e in A549 and HBE16 lung epithelial cells while the inhibitor suppressed protein expression. Interestingly, similar to miR-155, CD49d has been implicated as a poor prognostic marker for CLL.^11, 12^ To determine whether CSNK1G2 may regulate CD49d and/or CD49e levels, we proceeded to knock down *CSNK1G2* in CLL cells and see if this might lead to alteration in the levels of CD49d and CD49e. Indeed, when *CSNK1G2* mRNA was reduced by 38.9% using siRNA, the levels of CD49d and CD49e transcripts were increased by 43.2% and 35.8%, respectively (Figure 2c). These results suggest a link between miR-155, *CSNK1G2* and CD49d/e expression levels. It is conceivable that the positive modulatory effect of miR-155 on CD49d/e expression through downregulation of *CSNK1G2* may be one of the underlying mechanisms accounting for the association of high levels of miR-155 with less favorable clinical outcome.

We have also identified two putative miR-155 gene targets in CLL in our differential microarray screening: *ZNF652* and *KLF3*. Both of them harbor conserved miR-155-binding sites as predicted by Targetscan. Their interaction with miR-155 is also suggested by either luciferase reporter assay and/or quantitative reverse transcriptase-PCR results (data not shown). ZNF652 is a zinc finger protein that interacts with a ETO family protein CBFA2T3, a putative tumor suppressor in breast oncogenesis, to repress transcription.^13^ KLF3 is a member of the Kruppel-like factor (KLF) family of transcription factors that have diverse roles in cell proliferation, differentiation and apoptosis. Interestingly, KLF3 has been implicated to have important roles in B-cell biology.^14^ KLF3 expression is high in resting quiescent B cells but is reduced upon activation by B-cell receptor signaling, suggesting a role in maintaining cellular quiescence in B cells. KLF3 also appears to drive marginal zone B-cell differentiation. KLF3 is also the target gene in a common retroviral insertion site in B-cell lymphomas in mice, raising the possibility that alterations in KLF3 expression may promote B-cell lymphomagenesis.^15^

In summary, through global transcriptome profiling of CLL with differential miR-155 expression levels, we have successfully identified several putative targets of miR-155 in CLL. We have also performed additional studies to validate *CSNK1G2* as a miR-155 target in CLL and proposed a mechanism of how this interaction may account for the association of high miR-155 with clinical aggressiveness of CLL. Our study highlights the feasibility of this strategy in identifying potential gene targets of deregulated miRNAs in the natural context of various types of hematological malignancies. The biological functions of *CSNK1G2* and other putative targets of miR-155 (for example, *KLF3*) identified in this study may suggest a possible pathogenic role of these genes in CLL; additional studies to further investigate their contribution to CLL pathobiology may be warranted.

## Figures and Tables

**Figure 1:**
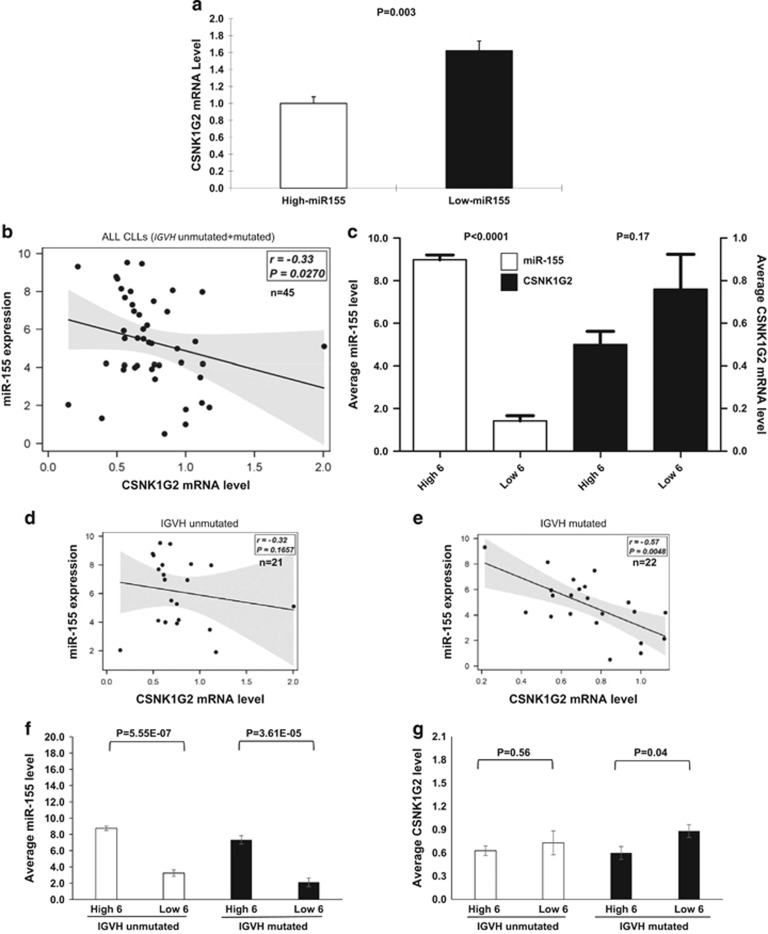
Expression correlation between hsa-miR-155 and *CSNK1G2* mRNA in CLL suggests a regulatory effect on the *IGVH*-mutated subset. (**a**) Expression of *CSNK1G2* mRNA in CLL patients (original cohort) subgrouped by high (*n*=11, marked by dots in Supplementary Figure S1A) and low (*n*=11, marked by asterisks in Supplementary Figure S1A) miR-155 expression. *CSNK1G2* mRNA levels were quantified by quantitative reverse transcriptase-PCR and normalized to *GUSB*. The average *CSNK1G2* mRNA expression in the high-miR-155 subgroup is arbitrarily set as one. The average *CSNK1G2* mRNA expression in the low miR-155 subgroup is 1.62 times that of the high miR-155 subgroup (*P*<0.05). (**b**) *CSNK1G2* mRNA levels are plotted against abundance of miR-155 in CLL/SLL B cells of 45 patients in the validation cohort. There was a weak but significant correlation (*P*=0.027) between miR-155 and *CSNK1G2* mRNA levels in these 45 CLLs. (**c**) Comparison of miR-155 and *CSNK1G2* expression levels between the subgroup of 6 CLL patients with the highest miR-155 levels (*n*=6, high 6) and the subgroup of 6 CLL patients with the lowest miR-155 levels (*n*=6, low 6). MiR-155 expression of the high subgroup was ~9 times that of the low (*P*<0.0001). The miR-155 low subgroup showed slightly higher *CSNK1G2* expression. However, statistical significance was not reached. (**d** and **e**) Correlation plots of miR-155 and *CSNK1G2* expression levels for the *IGVH*-unmutated and -mutated CLL subsets are shown. Although there is no significant correlation between miR-155 and *CSNK1G2* expression in the *IGVH*-unmutated CLLs, there is moderate negative correlation (*r*=−0.57, *P*=0.0048) in the *IGVH*-mutated subset. (**f** and **g**). MiR-155 and *CSNK1G2* mRNA levels of the top 6 (high 6) and bottom 6 (low 6) miR-155 expression in the *IGVH*-unmutated and -mutated CLL subgroups are indicated. Although there was no significance difference in the *CSNK1G2* mRNA expression in the top and bottom six miR-155 expression within the *IGVH*-unmutated group, *CSNK1G2* mRNA expression in the miR-155 low group is significantly higher (*P*=0.04) within the *IGVH*-mutated group.

**Figure 2:**
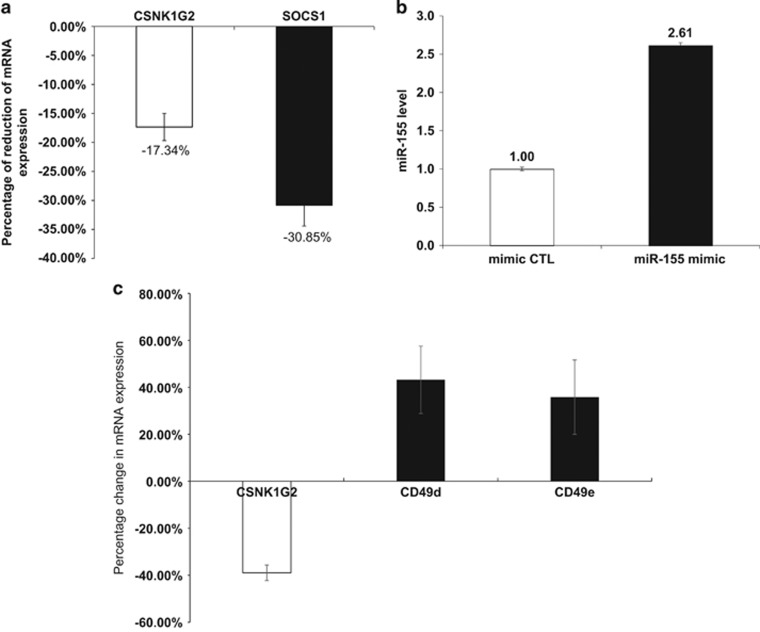
Enforced expression of miR-155 leads to a reduction in *CSNK1G2* mRNA and *CSNK1G2* knockdown results in increase of CD49d and CD49e expression levels in CLL cells. (**a** and **b**) Overexpression of miR-155 mimic in primary peripheral blood B cells of three *IGVH*-mutated CLL patients is accompanied by a decrease in *CSNK1G2* mRNA level by 17.3±2.3% compared with the control (mimic CTL). The mRNA expression of *SOCS1*, a known target of miR-155, is decreased by 30.9±3.6% (**a**). The average level of miR-155 upon overexpression of miR-155 mimic is 2.6 times that of the control (**b**). Mimic CTL, miRCURY LNA microRNA mimic Negative Control 4 (Exiqon). (**c**) Purified peripheral blood B cells from five *IGVH*-mutated CLL patients ware transfected with siRNA against *CSNK1G2*. The average reduction of *CSNK1G2* mRNA is 38.9%. This decrease in *CSNK1G2* is accompanied by an increase in CD49d and CD49e mRNA expression levels.
