# Supplementary figures and images for: MicroRNA-155 regulates casein kinase 1 gamma 2: a potential pathogenetic role in chronic lymphocytic leukemia
Source: Blood Cancer J. 2017 Sep 8;7(9):e606–. doi: 10.1038/bcj.2017.80 (PMC5709749; doi:10.1038/bcj.2017.80)

Fig. S1

A.

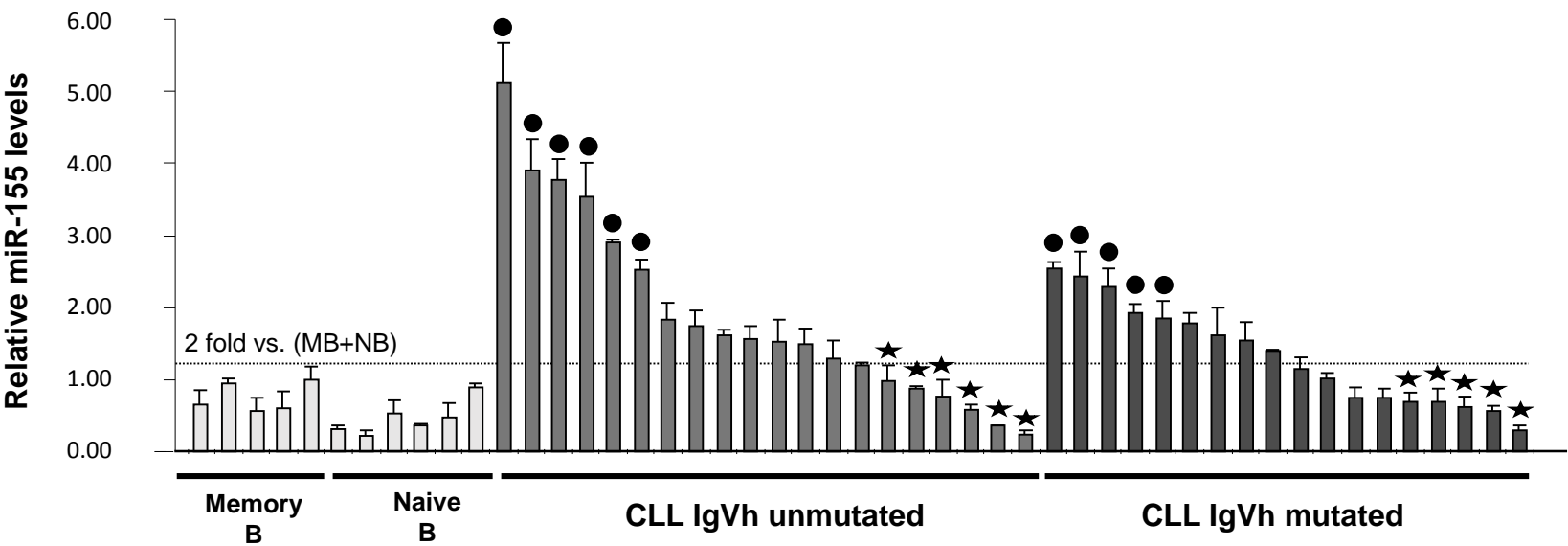

B.

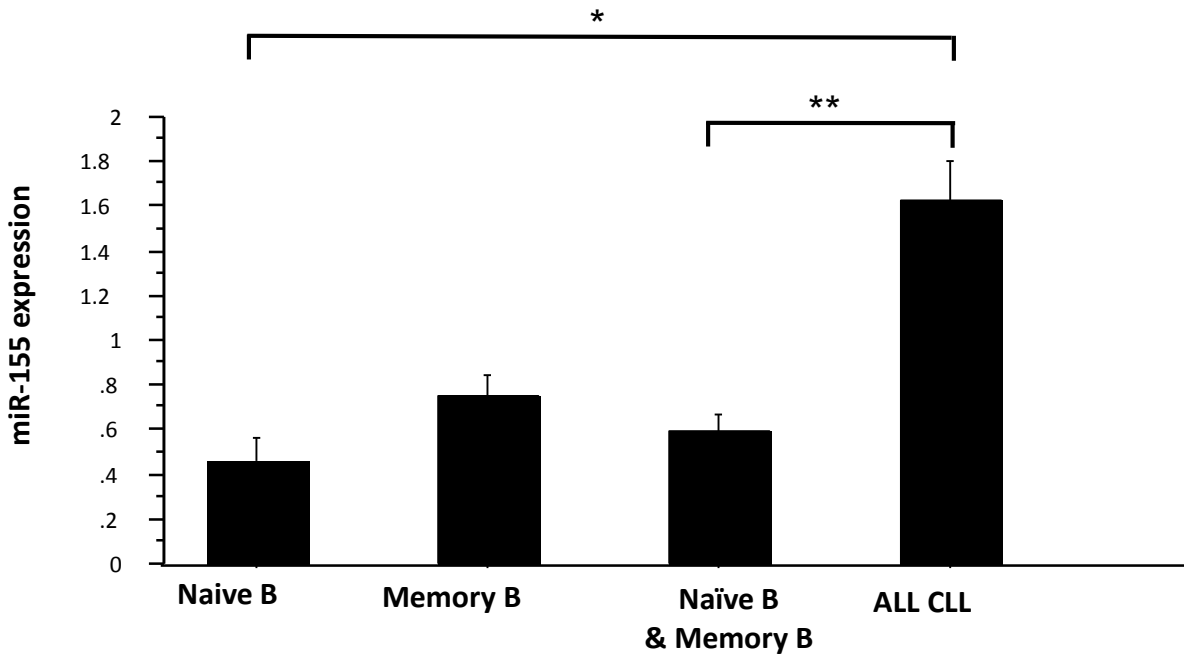

Supplement: Supplementary Figure S1 [file bcj201780x1.pdf]

Fig. S2

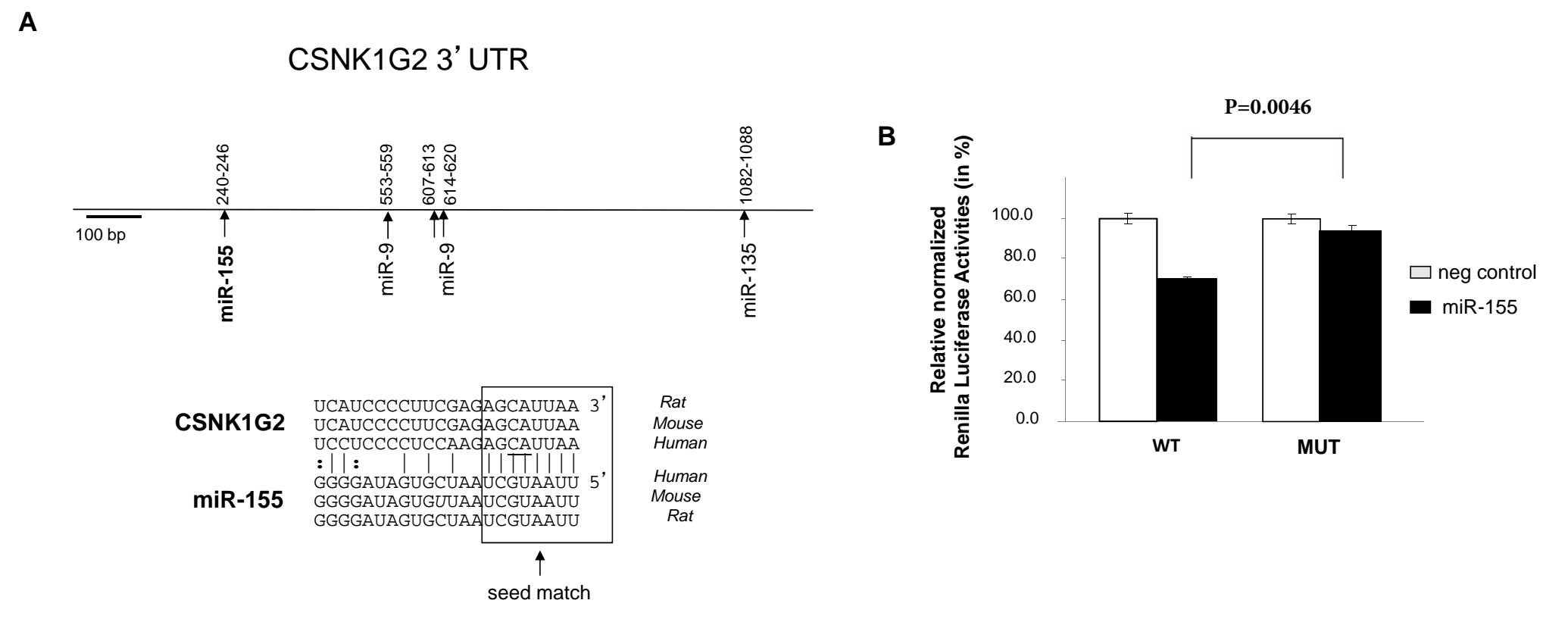

Supplement: Supplementary Figure S1 [file bcj201780x2.pdf]
