# Supplementary material for: MicroRNA-155 regulates casein kinase 1 gamma 2: a potential pathogenetic role in chronic lymphocytic leukemia
Source: Blood Cancer J. 2017 Sep 8;7(9):e606–. doi: 10.1038/bcj.2017.80 (PMC5709749; doi:10.1038/bcj.2017.80)
Supplement: Supplementary Table S1 [file bcj201780x3.pdf]

Table S1

	ID	Age	Gender	RAI stage	IgVH Mut	ZAP70	Cytogenetics	Vh	miR-155	Treatment status<br>A:Treatment Naïve (no prior or current treatment)<br>B: Prior treatment but not currently treated<br>C: Currently treated	Specimen
High miR-155	WT-14	77	M	IV	Um	POS	n/a	V7-4-1*02	5.1	C: CVP	PBL
	WT-24	62	M	III	Um	POS	NEG	V4-39*07	3.89	A	LN
	335	63	M	IV	Um	NEG	del 17p; del 6q	V4-31	3.76	B	PBL
	322	60	M	III or IV	Um	POS	13q14 del	V3-43	3.53	C: FR	PBL
	172	74	M	IV	Um	POS	11q22 del	V1-8	2.91	C: Enzastaurin	PBL
	WT-22	70	M	IV	Um	POS	13q14 del; 17p13 del	V7-4-1*02	2.52	A	PBL
	358	67	F	0 or I	M	POS	Normal	V3-7	2.54	A	PBL
	324	61	F	I or II	M	NEG	13q14 del	V3-23	2.29	C: Genitope anti-idiotype vaccine	PBL
	160	49	M	II	M	POS	del13q14.3; del11q22	V3-23	2.22	B	PBL
	351	72	F	0	M	NEG	13q14 del	V3-9	1.92	A	PBL
	320	59	M	II	M	NEG	13q14 del	V4-34	1.86	C: Genitope anti-idiotype vaccine	PBL
								Average	2.96		
Low miR-155	330	57	F	IIor III	Um	POS	Trisomy 12 **	V4-39*07	1.07	B	PBL
	WT-27	57	F	II	Um	POS	NA	V4-59*01	0.87	A	spleen
	300	70	M	NA	Um	NA	NA	V1-69*01	0.77	NA	PBL
	318	77	M	II	Um	POS	Trisomy 12	V2-5; 4-39	0.59	B	PBL
	WT-16	70	M	IV	Um	NEG	17p13 del	V3-33*01	0.36	A	PBL
	344	62	M	III or IV	Um	POS	Trisomy 12; 11q del	V4-31	0.23	B	PBL
	346	65	M	I or II	M	NEG	13q14 del	V1-18	0.69	B	PBL
	356	60	F	II	M	NEG	13q14 del	V3-30	0.69	A	PBL
	364	88	M	IV	M	POS	Tri 12, 13q14del, p53 del	V3-33*01	0.68	C: Campath	PBL
	166	53	M	II orIII	M	NEG	13q14 del	NA	0.62	A	PBL
	332	71	F	I or II	M	NEG	Trisomy 12	V4-31	0.3	C: Id-KLH vaccine	PBL
								Average	0.62		

** Prealence of trisomy 12 is higher in the low miR-155 subgroup (p=0.01)
