# Supplementary material for: MicroRNA-155 regulates casein kinase 1 gamma 2: a potential pathogenetic role in chronic lymphocytic leukemia
Source: Blood Cancer J. 2017 Sep 8;7(9):e606–. doi: 10.1038/bcj.2017.80 (PMC5709749; doi:10.1038/bcj.2017.80)
Supplement: Supplementary Table S2 [file bcj201780x4.pdf]

Table S2

List of down-regulated probes/genes identified by cDNA microarray comparing CLL with high and low miR-155 expressions

GeneBank ID	Gene name	Description	Fold Change	q-value(%)
AL521634	CoQ10A	coenzyme Q10 homolog A	0.71	0
**AL530441**	**CSNK1G2**	**casein kinase 1 gamma 2**	**0.79**	**0**
**NM_001319**	**CSNK1G2**	**casein kinase 1 gamma 2**	**0.77**	**0**
AI989996	KDM2B	lysine demethylase 2B	0.68	0
N32834	GLIPR1	GLI pathogenesis-related 1	0.45	3.16
**AA130132**	**KLF3**	**Kruppel-like facotr 3 (basic)**	**0.11**	**3.16**
BC006270	HECTD4	HECT domain E3 ubiquitin protein ligase 4	0.72	3.16
**NM_014897**	**ZNF652**	**Homo sapiens zinc finger protein 652**	**0.61**	**3.16**
AF289565	KLF3-AS1	KLF3 antisense RNA 1	0.74	3.16
**BF438116**	**KLF3**	**Kruppel-like facotr 3 (basic)**	**0.17**	**5.23**

Genes with predicted miR-155 binding sites are in bold.

FDR=false discovery rate, <0.05
