# Supplementary material for: MicroRNA-155 regulates casein kinase 1 gamma 2: a potential pathogenetic role in chronic lymphocytic leukemia
Source: Blood Cancer J. 2017 Sep 8;7(9):e606–. doi: 10.1038/bcj.2017.80 (PMC5709749; doi:10.1038/bcj.2017.80)
Supplement: Supplementary Table S3 [file bcj201780x5.pdf]

Table S3

S3A: Cohort 1

ID	Age	Gender	RAI stage	IgVH Mut	ZAP70	Cytogenetics	Vh	miR-155	Treatment status<br>A:Treatment Naïve (no prior or current treatment)<br>B: Prior treatment but not currently treated<br>C: Currently treated	Specimen
WT-14	77	M	IV	Um	POS	n/a	V7-4-1*02	5.1	C: CVP	PBL
WT-24	62	M	III	Um	POS	NEG	V4-39*07	3.89	A	LN
335	63	M	IV	Um	NEG	del 17p; del 6q	V4-31	3.76	B	PBL
322	60	M	III or IV	Um	POS	13q14 del	V3-43	3.53	C: FR	PBL
172	74	M	IV	Um	POS	11q22 del	V1-8	2.91	C: Enzastaurin	PBL
358	67	F	0 or I	M	POS	Normal	V3-7	2.54	A	PBL
WT-22	70	M	IV	Um	POS	13q14 del; 17p13 del	V7-4-1*02	2.52	A	PBL
324	61	F	I or II	M	NEG	13q14 del	V3-23	2.29	C: Genitope anti-idiotype vaccine	PBL
160	49	M	II	M	POS	del13q14.3; del11q22	V3-23	2.22	B	PBL
351	72	F	0	M	NEG	13q14 del	V3-9	1.92	A	PBL
320	59	M	II	M	NEG	13q14 del	V4-34	1.86	C: Genitope anti-idiotype vaccine	PBL
359	43	M	I	Um	NEG	Normal	V1-69	1.83	A	PBL
WT-11	74	F	II	M	POS	NA	V3-30*03	1.77	A	LN
WT-18	54	F	I	Um	POS	Trisomy 12	V1-69*12	1.75	A	LN
167	63	M	III	M	POS	Normal	V-34*01	1.74	A	PBL
337	82	M	II	M	NEG	Normal	NA	1.62	C: Rituxan	PBL
WT-20	73	F	IV	Um	POS	Tri 12, 13q14del, p53 del	V1-2*02	1.62	C: Campath (CD52ab)	PBL
165	74	M	IV	Um	POS	del 17p	NA	1.53	B	PBL
355	59	M	I	Um	Borderline/Indeterminate	11q del	NA	1.48	A	PBL
343	71	F	IV	Um	POS	del13q14.3; del11q22	NA	1.46	B	PBL
177	58	F	I	M	NEG	13q14 del	V7-4	1.39	A	PBL
329	63	F	II	Um	NEG	del13q14.3; del11q22	V1-2	1.28	C: Enzastaurin	PBL
WT-26	74	F	0	Um	NEG	Trisomy 12	V3-11*01	1.2	A	PBL
330	57	F	IIor III	Um	POS	Trisomy 12	V4-39*07	1.07	B	PBL
352	67	M	I	M	Neg	11q del	V3-30	1.06	B	PBL
WT-19	69	F	0	M	NEG	13q14.3	V4-34*01	1.02	A	PBL
WT-27	57	F	II	Um	POS	NA	V4-59*01	0.87	A	spleen
300	70	M	NA	Um	NA	NA	V1-69*01	0.77	NA	PBL
319	58	F	0/I	M	NEG	13q14 del	V3-53	0.74	A	PBL
325	52	F	I	M	NA	13q14 del	V4-34/V5-51	0.74	C: Genitope anti-idiotype vaccine	PBL
346	65	M	I or II	M	NEG	13q14 del	V1-18	0.69	B	PBL
356	60	F	II	M	NEG	13q14 del	V3-30	0.69	A	PBL
364	88	M	IV	M	POS	Tri 12, 13q14del, p53 del	V3-33*01	0.68	C: Campath	PBL
166	53	M	II orIII	M	NEG	13q14 del	NA	0.62	A	PBL
318	77	M	II	Um	POS	Trisomy 12	V2-5; 4-39	0.59	B	PBL
WT-16	70	M	IV	Um	NEG	17p13 del	V3-33*01	0.36	A	PBL
332	71	F	I or II	M	NEG	Trisomy 12	V4-31	0.3	C: Id-KLH vaccine	PBL
344	62	M	III or IV	Um	POS	Trisomy 12; 11q del	V4-31	0.23	B	PBL

S3B: Cohort 2

New ID	Age	Gender	RAI stage	IgVH Mut	ZAP70	Cytogenetics	Vh	miR 155	Treatment status<br>A:Treatment Naïve (no prior or current treatment)<br>B: Prior treatment but not currently treated<br>C: Currently treated	Specimen
416	58	M	II	Um	POS	Del 13q14.3	V1-69	9.53	C: Mid-ACP-196 C1D22	PBL
208	55	M	NA	Um	NA	NA	NA	9.47	NA	PBL
204	73	F	0	M	NEG	Del 13q14.3	V3-21	9.3	A	PBL
268	56	F	I	Um	NEG	Normal	V1-69	8.77	A	PBL
195	42	M	II	Um	NEG	Del 13q14.3; del 11q	V1-69	8.67	B	PBL
537	62	F	II	M	NEG	Del 13q14.3	V3-30	8.14	C: Mid-THRIL	PBL
336	55	F	II	Um	POS	Del 13q14.3	V3-48	8.05	B	PBL
629	61	M	II	Um	NEG	del 17p; del 13q14.3; del 11q	V4-4	7.99	B	PBL
425	56	M	I	Um	NEG	Del 13q14.3	V1-69	7.97	A	PBL
144	78	F	I	Um	NEG	del 17p; del 13q14.3	V1-69	7.68	C: Mid-GS-1101-117	PBL
196	56	F	0	M	NEG	Del 13q14.3	V3-23	7.48	A	PBL
223	52	M	I	Um	NEG	Normal	V3-53	7.3	A	PBL
426	71	F	III	Um	NEG	Del 13q14.3; del 11q	V3-23	6.96	B	PBL
205	78	F	0-I	Um	NEG	del 17p; del 13q14.3	V3-11	6.93	A	PBL
583	58	F	II	M	POS	Del 13q14.3	V3-43	6.77	C: Mid-THRIL	PBL
457	63	M	II	M	NEG	Normal	V3-7	6.22	A	PBL
184	38	M	0	M	POS	Del 13q14.3	V1-18	6.02	A	PBL
217	58	M	I	M	POS	Del 13q14.3	NA	5.94	B	PBL
215	83	M	IV?	M	POS	Del 13q14.3	V6-1	5.53	B	PBL
234	60	F	0 or I	M	NEG	Del 13q14.3	V2-5	5.53	A	PBL
235	57	F	IV	Um	NEG	Del 13q14.3; tri12? prior	V3-53	5.51	A	PBL
197	78	M	NA	M	NA	NA	NA	5.32	NA	PBL
106	35	M	I	Um	POS	Del 13q14.3; del 11q	V3-74	5.26	A	PBL
464	69	M	I-II	Um	NEG	del 17p; trisomy 12	V1-69	5.1	C: Mid-GS-1101-116	PBL
188	75	F	0	M	NEG	Del 13q14.3	V3-30	4.98	A	PBL
677	63	M	I	M	NEG	Del 13q14.3	NA	4.25	A	PBL
236	61	M	0	M	NA	NA	V4-34	4.2	A	PBL
455	62	M	IV	Um	NEG	del 6q	V3-33	4.15	C: Mid-GS-1101-116	PBL
362	66	M	0	M	NA	Trisomy 12	VH40-4/ V3-23	4.14	B	PBL
253	74	M	IV	Um	POS	Del 17p, Del 13q14.3, Tri12	V4-34	4.11	A	PBL
192	82	F	NA	M	NEG	Normal	NA	4.1	B	PBL
353	67	F	0	M	NEG	Del 13q14.3	V3-33	4.06	B	PBL
207	65	M	I? NA?	Um	POS	Del 13q14.3	V1-18	3.99	B	PBL
406	66	M	IV	Um	POS	Del 17p, Del 13q14.3, Tri12, Del 11q	V3-23	3.9	C: Mid-CAL-101	PBL
692	83	M	IV	M	NEG	Del 13q14.3	V4-34	3.88	B	PBL
191	87	F	I	M	NA	NA	NA	3.48	NA	PBL
375	62	F	II	M	NEG	Del 13q14.3	V3-23	3.38	A	PBL
281	76	F	0	M	NEG	Del 13q14.3	V4-59	2.13	A	PBL
227	70	M	II	Um	POS	Del 17p?, Del 13q14.3, Tri12	V4-4	2.04	B	PBL
198	87	M	IV	Um	NEG	Del 11q; trisomy 12	V1-69	1.89	A	PBL
585	48	M	II	M	NEG	Del 13q14.3	V2-5	1.79	B	PBL
332	71	F	II	M	NEG	Trisomy 12	V4-31	1	B	PBL
201	72	M	II	M	NA	Trisomy 12	NA	0.5	A	PBL
