# Supplementary material for: MicroRNA-155 regulates casein kinase 1 gamma 2: a potential pathogenetic role in chronic lymphocytic leukemia
Source: Blood Cancer J. 2017 Sep 8;7(9):e606–. doi: 10.1038/bcj.2017.80 (PMC5709749; doi:10.1038/bcj.2017.80)
Supplement: Supplementary Figure Legend [file bcj201780x6.docx]

**Supplementary information**

**Figure S1. MiR-155 expression in patient and control samples.**

**A.** miR-155 levels were measured by quantitative real-time PCR from 38 B-cell purified CLL samples, 5 sets of naïve and 6 sets of memory B cells isolated from normal hyperplastic tonsils. Expression in each sample is represented by a vertical bar. The data are shown as means (±S.E.) of 3 independent measurements. The level of miR-155 that corresponds to 2 fold of the average expression of the naïve and memory B cells was marked by a horizontal line. Cases with highest and lowest miR-155 levels in each *IGVH* subgroup were selected for cDNA microarray analysis (indicated by dots and asterisks, respectively).

**B.**  The average (±S.E.) of miR-155 levels in CLL cases as an entire group was compared to naïve and memory B cells. * and ** indicate statistical significance (p=0.01 and p<0.005, respectively).

**Figure S2. *CSNK1G2* contains conserved functional miR-155 target sites in its 3’ UTR.**

**A.** The positions of the predicted miR-155 target site, along with several other predicted miRNA sites in the *CSNK1G2* 3’ UTR are indicated. The numbers are relative to the beginning of the 3’ UTR. Pairing between miR-155 and its putative target site in *CSNK1G2* 3’UTR is shown. The highly conserved 8 bp “seed” pairing is highlighted by open box. The nucleotides of the miR-155 target site that were mutated to disrupt the “seed” pairing are underlined.

**B.** Reporter plasmids harboring *CSNK1G2* 3’UTR with wild-type sequence (WT) or point mutations in the miR-155 target site (MUT) were co-transfected into 293T cells with 100ng pcDNA3.BIC/miR-155 plasmid (miR-155) or with 100 ng pcDNA3 plasmid (negative control). Luciferase activities (in triplicates) were measured 24 hours after transfection. *Renilla* Firefly luciferase activities were normalized against firefly luciferase activities, and mean normalized *Renilla* luciferase activities (±S.E.) were determined and expressed relative to control values.

**Table S1. Clinical and cytogenetics characteristics of CLL patients sub-grouped by high and low miR155.**

We selected the 6 top-ranked and 5 bottom-ranked in each CLL subgroup (Fig 1A; *IGVH* unmutated or *IGVH* mutated; total 22 cases) for global cDNA expression analysis using Affymetrix Human Genome U133 Plus 2.0 Array GeneChip (Table 2). The microarray data was deposited at Gene Expression Omnibus (accession number GSE29605).

**Table S2: List of 8 genes differentially down regulated in high miR-155 CLL vs. low miR-155 CLL.**

Global cDNA expression analysis using Affymetrix Chip were compared between CLL with high miR-155 expression and CLL with low miR-155 expression using SAM analysis (5% false discovery rate). A gene is likely to be a true target for miR-155 in CLL if it is down-regulated in CLL with high miR-155 levels relative to CLL with low miR-155 levels, and if the 3’ UTR of this gene contains predicted miR-155 binding sites (figure 2A).

**Table S3: Clinicopathologic characteristics of CLL patients in the two cohorts.**

The age, gender, RAI stage, cytogenetics, IGV gene usage, ZAP70 expression, treatment status at the time of specimen collection, specimen type and miR-155 expression are listed in the table for cohort 1 (38 patients, see Fig. S1) and cohort 2 (43 patients, Fig. 1D and E).

All patients’ samples were procured according to the protocols approved by the Institutional Review Board at Weill Cornell Medicine. Informed consent was obtained from all patients.
